# Clinical and genetic analyses reveal novel pathogenic *ABCA4* mutations in Stargardt disease families

**DOI:** 10.1038/srep35414

**Published:** 2016-10-14

**Authors:** Bing Lin, Xue-Bi Cai, Zhi-Li Zheng, Xiu-Feng Huang, Xiao-Ling Liu, Jia Qu, Zi-Bing Jin

**Affiliations:** 1 The Eye Hospital of Wenzhou Medical University, The State Key Laboratory Cultivation Base and Key Laboratory of Vision Science, Ministry of Health, Wenzhou 325027, China

## Abstract

Stargardt disease (STGD1) is a juvenile macular degeneration predominantly inherited in an autosomal recessive pattern, characterized by decreased central vision in the first 2 decades of life. The condition has a genetic basis due to mutation in the *ABCA4* gene, and arises from the deposition of lipofuscin-like substance in the retinal pigmented epithelium (RPE) with secondary photoreceptor cell death. In this study, we describe the clinical and genetic features of Stargardt patients from four unrelated Chinese cohorts. The targeted exome sequencing (TES) was carried out in four clinically confirmed patients and their family members using a gene panel comprising 164 known causative inherited retinal dystrophy (IRD) genes. Genetic analysis revealed eight *ABCA4* mutations in all of the four pedigrees, including six mutations in coding exons and two mutations in adjacent intronic areas. All the affected individuals showed typical manifestations consistent with the disease phenotype. We disclose two novel *ABCA4* mutations in Chinese patients with STGD disease, which will expand the existing spectrum of disease-causing variants and will further aid in the future mutation screening and genetic counseling, as well as in the understanding of phenotypic and genotypic correlations.

Stargardt disease (STGD1: OMIM #248200) is an inherited macular dystrophy leading to progressive loss of central vision in early childhood, accounting for approximately 7% of all retinal dystrophies and affecting about 1 in 10,000 individuals^1^. Most affected individuals maintain full peripheral visual function albeit with central scotomas of miscellaneous sizes. A progressive decrease in central visual acuity occurs throughout life with values approaching 0.1 or worse in the final stages of the disease. However, fundus examination is often normal in the early stage, even when patients already experience a significant vision loss. Later on, typical fundus manifestations arise, including mottling or atrophy of the RPE, bull’s eye maculopathy, beaten-bronze macular appearance, and an accumulation of discrete “pisciform” flecks around the macula and/or in the midperipheral retina^2^. The fluorescein angiography (FA) displays a “dark-choroid” sign in up to 80% of patients^3^,^4^.

Histologically, Stargardt disease links tightly with a massive deposition of lipofuscin content in the retinal pigment epithelium (RPE), failure in toxic substances removing and significant loss in photoreceptor cells^5^,^6^,^7^. Lipofuscin deposits consist of a heterogeneous mixture containing oxidized lipids, diretinoids, and other constituents derived from the incomplete degradation of photoreceptor outer segments (POS)^8^. As a lipid transporter specifically located in the rim of the photoreceptor discs in the outer segments of rods and cones and potentially facilitates the removal of toxic retinal compounds from photoreceptors, the ABCR gene (now called ABCA4) has been identified as the causative gene. This photoreceptor-specific gene is localized to chromosome 1p21–22, and there are currently over 800 disease-associated variants for the *ABCA4* gene, more than half of which have been detected only once^9^. A major component of lipofuscin, Di-retinoid-pyridinium-ethanolamine (A2E), is formed when the ABCA4 rim protein is missing or dysfunctional. As a hallmark of cellular degeneration, A2E eventually accumulates in the cells of the RPE, resulting in RPE cell death and probably secondary loss of photoreceptors^10^,^11^.

In spite of the fact that a large number of mutations in the *ABCA4* gene have been reported worldwide, there are a few investigations of Chinese patients with Stargardt disease^12^,^13^. We herein describe the clinical and genetic features of Stargardt patients from four unrelated Chinese cohorts, and reveal eight *ABCA4* mutations in all of the four pedigrees, adding two novel mutations to the existing spectrum of disease-causing variants in the *ABCA4* gene.

## Materials and Methods

### Subjects and clinical assessment

The protocol in this study was approved by the Ethics Committee of The Eye Hospital of Wenzhou Medical University. Written informed consent was obtained from each participant, and all methods were in accordance with the Declaration of Helsinki. Our study recruited four clinically confirmed unrelated patients with Stargardt disease and 300 healthy controls. All of the four probands and their available family members were evaluated by a retina specialist. Full ophthalmologic examinations were performed on affected members, including visual acuity measurement, slit-lamp examination, dilated fundus photography, fundus fluorescein angiography (FFA), color vision testing, optical coherence tomography (OCT). In the 300 normal matched controls, all individuals underwent an eye assessment and no signs of any eye disease were observed. Venous blood samples were collected from all subjects in EDTA Vacutainers and preserved frozen.

### Targeted exome sequencing and in-depth bioinformatics analyses

To generate the Illumina paired-end libraries, we extracted a minimum of 3 μg of genomic DNA from each blood sample, sheared it into fragments by nebulization, repaired the DNA fragments with an ‘A’ ligated to the 3′ end, and the ultimate 350- to 400-base pair products were selected for PCR amplified after Illumina adapters were ligated to the fragments. Biotinylated single-strand DNA capture probes designed to potentially tile along 164 known causative inherited retinal dystrophy (IRD) genes were hybridized in solution with the target library. Illumina sequencing was performed in the light of a previous study^14^. Sequence reads were aligned to the reference human genome (hg19) using the SOAPaligner program (http://soap.genomics.org.cn/). Single-nucleotide polymorphisms (SNPs) were first identified using the SOAPsnp program (http://soap.genomics.org.cn/soapsnp.html) after filtering polymerase chain reaction duplicates by the Picard software. Insertions or deletions (InDels) were identified using BWA (http://bio-bwa.sourceforge.net/) and GATK programs (https://www.broadinstitute.org/gatk/). The candidate SNPs and InDels were annotated using the following five databases, including dbSNP137 (http://hgdownload.cse.ucsc.edu/goldenPath/hg19/database/snp137.txt.gz.), HapMap Project (ftp://ftp.ncbi.nlm.nih. gov/hapmap), 1000 Genome Project (ftp://ftp.1000genomes.ebi.ac.uk/vol1/ftp), YH database (http://yh.genomics.org.cn/), and Exome Variant Server (http://evs.gs.washington.edu/EVS/).

### Variants validation

After the initial filtration, the remaining variants were performed with Sanger sequencing to determine whether any of them co-segregated with the disease phenotype in the four families enrolled in our study. DNA sequences were acquired from the UCSC Genome Browser. Sequencing data were compared with those of the 300 normal matched controls and the Human Genome database.

### In silico analyses

In silico predicting online available programs provided information on the pathogenicity of the variants. The splice-site effect was assessed using the BDGP (http://www.fruitfly.org/seq_tools/splice.html) and ASSP programs (http://wangcomputing.com/assp/index.html). Missense variants were evaluated with PolyPhen-2 (http://genetics.bwh.harvard.edu/pph2/), SIFT (http://sift.jcvi.org/www/SIFT_enst_submit.html), and MutationTaster (http://www.mutationtaster.org/). Predicted crystal structures of the wild-type and mutant proteins were obtained using Phyre2 (http://www.sbg.bio.ic.ac.uk/phyre2/html/page.cgi?id=index)^15^ and displayed by PyMol software (Version 1.5).

## Results

### Subjects and phenotyping

**Figure 1:**
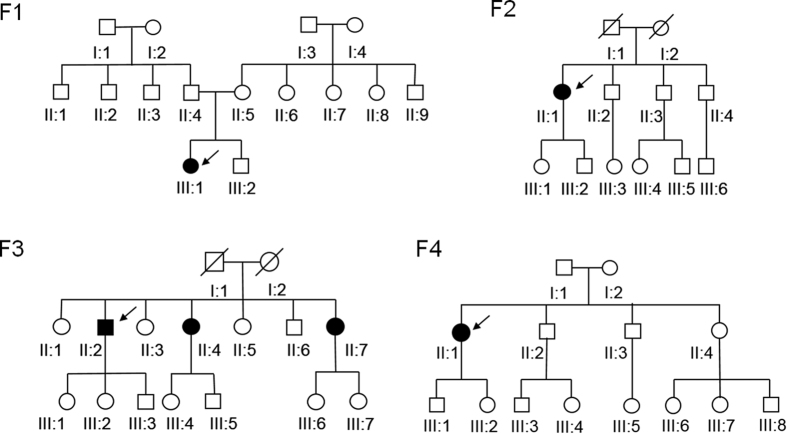
Pedigrees of four Chinese families with Stargardt disease. Squares and circles represent males and females, respectively, and darkened symbols indicate the affected members. Deceased family members are noted with a diagonal line. The patient below the arrow is the proband of each family.

**Figure 2:**
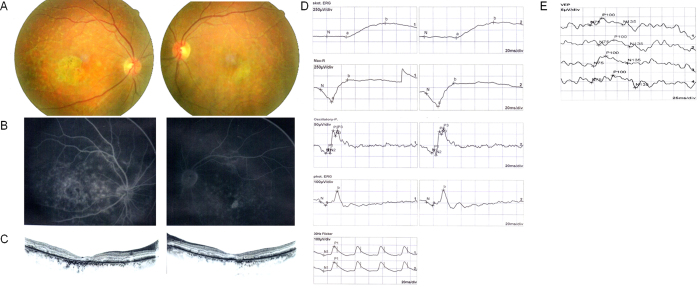
Representative photographs of proband F2:II:1. (**A**) Fundus photographs showing a central atrophic lesion with flecks around the macula. (**B**) FA images showing fluorescence blocking caused by the pigment mottling in the macular, hyperfluorescent flecks extended to the midperipheral retina, and improved visualization of the retinal vessels with normal caliber, which became rather obvious over the dark, non-fluorescent and high-contrast choroid. (**C**) Macular OCTs showing a reduced thickness of the attenuated retina and an altered reflectivity in the choroid, RPE and the outer segments of the photoreceptors in both eyes. (**D**) Full field ERG showing normal ERG values. (**E**) Pattern VEP showing an increase in the latency of P100.

Four unrelated patients were clinically diagnosed with Stargardt disease and their detailed family histories were obtained (Table 1, Fig. 1). All the affected members complained of an early-onset markedly decreased vision acuity in both eyes along with an increasing difficulty in dark adaptation and a variable loss in color vision. On fundus examination, typical presentations were observed, including some pigment mottling, beaten-bronze macular appearance, and yellow-white flecks around maculae, while few pigmented bone spicules were seen in the retinal periphery (Fig. 2A). The fluorescein angiogram displayed fluorescence blocking due to the pigment mottling in the macular, hyperfluorescent flecks extended to the midperipheral retina, and a characteristic ‘dark choroid’ (Fig. 2B). The macular OCT showed enhanced choroidal reflectivity and thinning of the retinal outer layers, with hyper-reflective deposits localized between the RPE layer and the outer segments of the photoreceptors (Fig. 2C). Full field electroretinography (ERG) of both eyes was normal in all five recordings, including response to dim stimulation in dark adaptation (scotopic rod response), response to a bright stimulus in dark adaptation (scotopic combined rod-cone response), oscillatory potentials, response to a bright stimulus in light adaptation (photopic single-flash cone response), and response to a flickering stimulus in light adaptation (photopic 30-Hz flicker cone response), suggestive of group 1 STGD (Fig. 2D). Pattern visual evoked potential (VEP) uncovered the severity of the disease due to a prolongation of P100 latency (Fig. 2E). The fundus pictures of the other 3 probands are provided in the Supp. Fig 1.

### Mutations identified by TES

**Figure 3:**
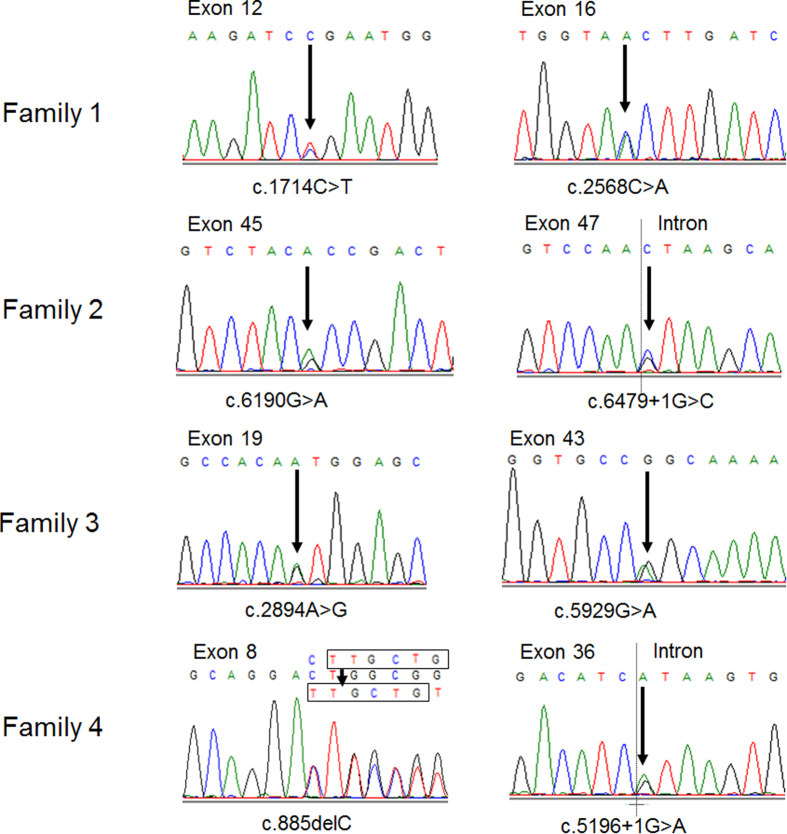
Sequencing results of the affected members in this study. Eight mutations of *ABCA4* gene were identified in the four Chinese pedigrees, with each family harboring two different mutations.

**Figure 4:**
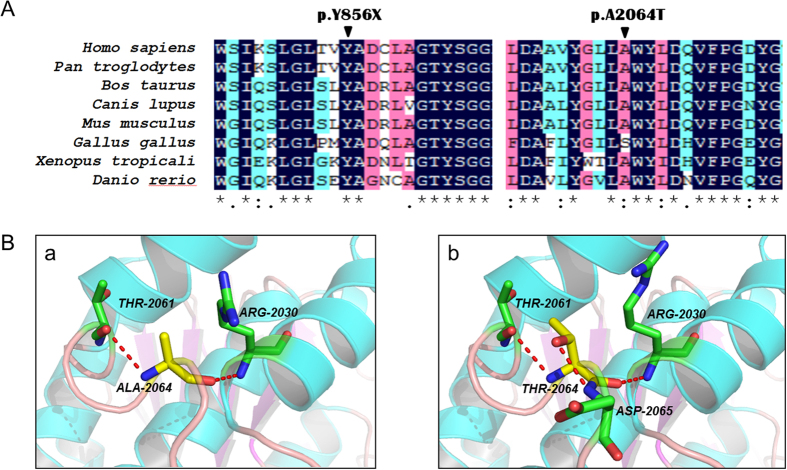
Genetic analyses of the two novel mutations identified in the *ABCA4* gene. (**A**) Multiple-sequence alignment of ABCA4 protein from different species showing the conserved amino acid residues (Tyrosine 856 and Alanine 2064). (**B**) Predicted crystal structures of wild-type (a) and mutant (b) ABCA4 protein showing an amino-acid mutation of Alanine to Threonine at a conserved position 2064.

A panel comprising 164 known IRD-related genes from RetNet and OMIM was used for TES. We discovered a total number of 650 variants in the samples. To identify pathogenic variants, we analyzed these variants through the approach of subjecting them to an analytical pipeline for variant calling, annotation and filtration, and finally identified two novel mutations in addition to six previously reported mutations in *ABCA4* gene (Fig. 3). In family 1, the proband F1:III:1 harbored two heterozygous nonsense mutations (c.2568C > A, p.Y856X; c.1714C > T, p.R572×^16^). To the best of our knowledge, this is the first report of the Y856X mutation in *ABCA4*. In family 2, a novel missense mutation (c.6190G > A, p.A2064T) and a known splicing mutation (c.6479 + 1G > C)^17^ were identified in the proband F2:II:1. The A2064T mutation occurred at a residue that is evolutionarily highly conserved (Fig. 4A). In family 3, the proband F3:II:2 and two of his siblings F3:II:5 and F3:II:7 shared two previously reported missense mutations (c.2894A > G, p.N965S^18^; c.5929G > A, p.G1977S^19^) in *ABCA4*. In family 4, in addition to a splicing mutation (c.5196 + 1G > A)^20^, we discovered a deletion mutation (c.885delC, p.L296CfsX4)^17^ in the proband F4:II:1, which was predicted to cause a frameshift at codon 296 and stop the open reading frame at codon 299, generating a prematurely truncated ABCA4 protein. These mutations were not present in any of the unaffected family members or 300 normal controls. The rest of the coding regions and adjacent intronic areas did not show any sequence changes. Co-segregation testing in all four families were carried out. The results showed that the all these four probands harbored compound heterozygous mutations (Supp. Fig. 2).

### Pathogenic assessment and structural modeling

Five of the detected mutations were predicted to affect correct RNA maturation either by disrupting conserved splice sites (c.6479 + 1G > C and c.5196 + 1G > A), introducing a frame shift (p.L296CfsX4) or by the introduction of a premature stop codon (p.R572X and p.Y856X). In silico predictions supported a pathogenic effect of all these mutations. To access the pathogenicity of the three missense mutations, we evaluated the predicted amino acid substitutions for conservation based on three types of online predictive software (Table 1). All substitutions changed highly conserved amino acids and were rated as “not tolerated” using SIFT. Structural modeling revealed the generation of a novel hydrogen bond between the mutated threonine at residue 2064 and asparagine at residue 2065 (Fig. 4B).

## Discussion

Stargardt disease is the most common cause of childhood recessively inherited macular degeneration. An adenosine triphosphate-binding cassette, sub-family A, member 4 (ABCA4) gene, has been identified as the causative gene. In spite of the fact that some dominant pedigrees have been uncovered, the vast majority of reported STGD1 cases are autosomal recessive. Thus it is common for a patient to be the only affected member in a family. It is therefore difficult to determine the pathogenicity of rare variants. To widen the knowledge in this field, we have studied four families with eight different *ABCA4* mutations.

Among the three missense mutations found in family 2 and family 3, the p.A2064T mutation had not been reported previously and thus was ascertained to be novel. To predict the pathogenicity, we constructed a crystal structure of mutant ABCA4 protein for this novel mutation, and compared it with the wild-type one. Because the three-dimensional protein structure of ABCA4 has not been available yet, we used LptB (PDB ID: 4P31) as the template to predict the likely structure impact of p.A2064T substitution on ABCA4 protein with PyMOL, based on their similar functions as ATP-binding proteins^21^. The ATP-binding cassette (ABC) systems comprise a broad and heterogeneous group of proteins that specialized in the active transport of various substrates across all domains of life^22^,^23^. Many of these transporters share a common architecture of two transmembrane domains (TMDs) and two cytoplasmic nucleotide-binding domains (NBDs), which couple the energy of ATP binding and hydrolysis to the transport across cellular membranes, against a concentration gradient. The modeled structure of mutated protein was composed of chains A and B, with the residue ALA-2064 located at chain B. The p.A2064T mutation would induce an additional hydrogen bond between the mutated threonine at residue 2064 and asparagine at residue 2065, which indicated that this substitution would significantly interfere with the interaction among distinct amino acids and thus transform its three-dimensional structure. The other two missense mutations p.N965S and p.G1977S were respectively first described in Danish and Spanish people. As the Danish founder mutation, the p.N965S mutation was found to account for 16.2% of the pathogenic alleles among patients of Danish origin^24^.

The rest five other variants were interpreted as null mutations: two were nonsense mutations (p.R572X and p.Y856X); two were splice-site mutations (c.6479 + 1G > C and c.5196 + 1G > A); and one was frameshift variant (p.L296CfsX4). Our data suggested that each of them issued in a truncated protein that was unable to function normally *in vivo* and was supposed to cause a severe reduction in ATP-binding capacity.

A wealth of our knowledge about ABCA4 function derives from genetic knockout experiments in genetically engineered mice. In terms of retinal appearance and photoreceptor structure, either homozygous or heterozygous mice for *ABCA4* remain normal. From a biochemical standpoint, however, *ABCA4*-knockout mice fail to transport N-retinylidene-phosphatidylethanolamine (NRPE) and phosphatidylethanolamine (PE) across the outer segment disc membranes to the cytoplasmic leaflet following photobleaching of opsins^10^,^25^. The animal models demonstrate that *ABCA4* is not required for normal photoreceptor structure or morphogenesis, but plays a metabolic role that facilitates the removal of retinoid byproducts from disc membranes after photobleaching of rhodopsin, preventing the accumulation of potentially toxic retinoid compounds in the subcellular space. These experiments in the *ABCA4*-knockout mice support the pathophysiological model of human disease. Fundus flecks found in proband F2:II:1 translate the accumulation of lipofuscin in the RPE. The fluorescence blocking in the macular, hyperfluorescent flecks extended to the midperipheral retina and the characteristic ‘dark choroid’ seen in FA relate to excessive lipofuscin accumulation in the RPE and low level RPE metabolic activity which normally underlies local atrophy with secondary photoreceptor loss. This low level of activity could explain the prolonged dark adaptation found in our Stargardt patients and could also explain the residual vision. Atrophic changes in the photoreceptors and a disruption of the foveal RPE are demonstrated on the OCT image of the proband F2:II:1. Lipofuscin deposits can be detected within the parafoveal RPE. Determining the status of the photoreceptor layer on OCT may provide an assessment of the central visual function.

In conclusion, we describe the clinical and genetic features of four Chinese Stargardt families, with different family members harboring various combinations of mutations. Though a visual acuity test, ophthalmoscopic examination, FA, and OCT are undeniably important for the early detection of Stargardt disease, molecular testing is necessary for a reliable diagnosis^26^. We reveal the identification of eight mutations in the *ABCA4* gene, including two novel mutations, and determine the pathogenicity of the rare variants, which may be useful in prognostic counseling and when it comes to choosing cases for future gene therapy.

## Additional Information

**How to cite this article**: Lin, B. *et al*. Clinical and genetic analyses reveal novel pathogenic *ABCA4* mutations in Stargardt disease families. *Sci. Rep.* **6**, 35414; doi: 10.1038/srep35414 (2016).

## Supplementary Material

Supplementary Information

## Figures and Tables

**Table 1:** *ABCA4* mutations identified in the study.

Patient	Age	BCVA	Exon	Variation				PolyPhen-2	Mutation taster	SIFT	ExAC	References
				Nucleotide	Protein	Status	Type					
F1:III:1	9	0.15/0.15	12	c.1714C > T	p.R572X	Het	Nonsense	—	—	—	Rare	12
			16	c.2568C > A	p.Y856X	Het	Nonsense	—	—	—	Novel	This study
F2:II:1	44	0.06/0.06	45	c.6190G > A	p. A2064T	Het	Missense	PD(0.988)	DC(0.999)	D(0.016)	Novel	This study
			47	c.6479 + 1G > C	p.?	Het	Splicing	—	—	—	Rare	13
F3:II:2	49	FC/FC	19	c.2894A > G	p.N965S	Het	Missense	PD(0.999)	DC(0.999)	D(0.012)	Rare	14
			43	c.5929G > A	p.G1977S	Het	Missense	PD(1.000)	DC(0.999)	D(0.000)	Rare	15
F4:II:1	45	FC/FC	8	c.885delC	p.L296CfsX4	Het	Frameshift	—	—	—	Rare	13
			36	c.5196 + 1G > A	p.?	Het	Splicing	—	—	—	Rare	16

BCVA, best corrected visual acuity; FC, fingers count; Het, heterozygous; PD probably damaging; DC, disease causing; D, damaging. Rare, MAF < 0.01.
