# Supplementary material for: Clinical and genetic analyses reveal novel pathogenic ABCA4 mutations in Stargardt disease families
Source: Sci Rep. 2016 Oct 14;6:35414. doi: 10.1038/srep35414 (PMC5064356; doi:10.1038/srep35414)
Supplement: Supplementary Information [file srep35414-s1.docx]

**Clinical and genetic analyses reveal novel pathogenic *ABCA4* mutations in** **Stargardt disease families**

Bing Lin^#^, Xue-Bi Cai^#^, Zhi-Li Zheng, Xiu-Feng Huang, Xiao-Ling Liu, Jia Qu, Zi-Bing Jin^*^

The Eye Hospital of Wenzhou Medical University, The State Key Laboratory Cultivation Base and Key Laboratory of Vision Science, Ministry of Health, Wenzhou 325027, China.

#These authors contributed equally to this work.

***Correspondence**: Dr. Zi-Bing Jin, The Eye Hospital of Wenzhou Medical University, The State Key Laboratory Cultivation Base and Key Laboratory of Vision Science, Ministry of Health, Wenzhou 325027, China. E-mail: [jinzb@mail.eye.ac.cn](mailto:jinzb@mail.eye.ac.cn). Tel/fax: +86-577-88067926.

**Competing interests**

The authors have declared that no competing interests exist.

**The Supplementary information included Supplementary Figure 1, 2.**


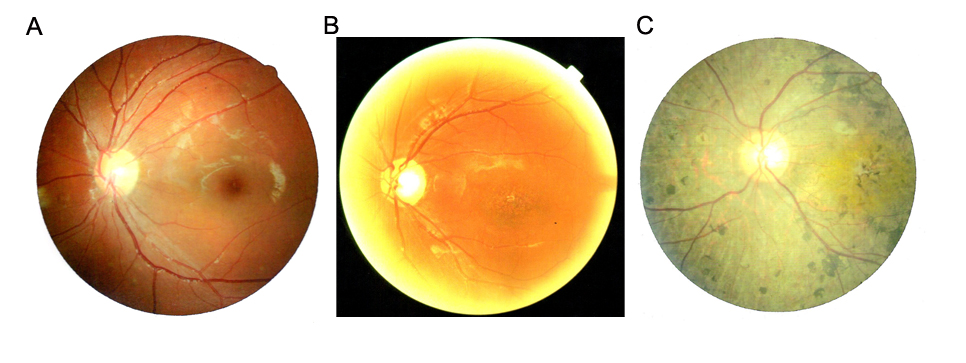


**Supplementary Figure 1. The fundus of the three probands.** (A-C), the fundus of patients F1:III:1, F3:II:2 and F4:II:1.


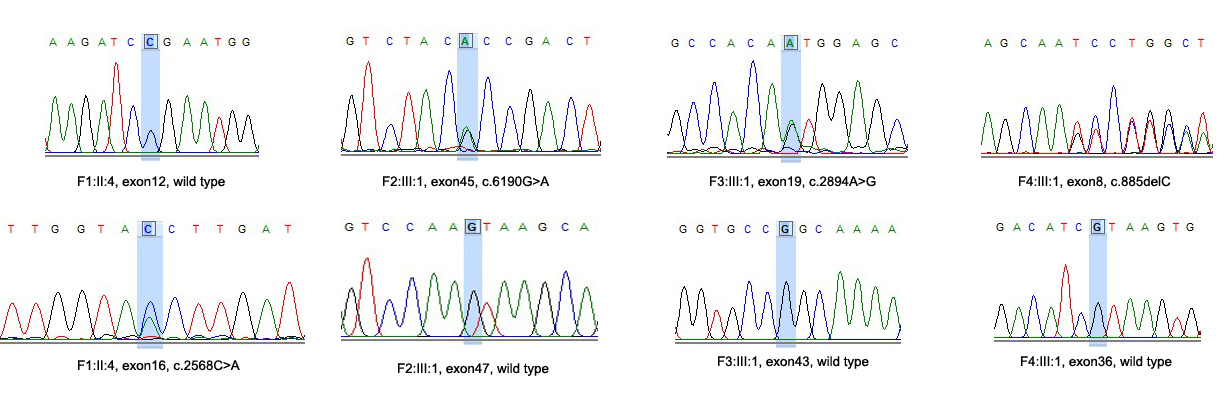


**Supplementary Figure 2. The co-segregation testing in four families.**
